# Rare case of Primary Pulmonary Extranodal Non-Hodgkin’s Lymphoma in a Patient with Sjogren’s Syndrome: Role of FDG-PET/CT in the Initial Staging and Evaluating Response to Treatment

**DOI:** 10.4274/Mirt.76

**Published:** 2012-12-20

**Authors:** Gonca Bural, Judith Joyce, James Ohr

**Affiliations:** 1 University of Pittsburgh Medical Center (UPMC), Radiology, Division of Nuclear Medicine, Pittsburgh, USA; 2 University of Pittsburgh Medical Center (UPMC), Department of Internal Medicine, Hematology and Oncology, Hillman Cancer Center, Pittsburgh, USA

**Keywords:** 18F-FDG, positron-emission tomography/computed tomography, marginal zone B-Cell lymphoma, Sjogren’s syndrome

## Abstract

A 64-year old woman with a long standing Sjogren’s syndrome was undergoing evaluation for renal transplant surgery when two pulmonary opacities were detected on chest CT. Subsequent biopsy revealed extranodal marginal B-cell non-Hodgkin’s lymphoma (NHL). An FDG-PET/CT scan was then performed which demonstrated isolated FDG avid pulmonary involvement. After therapy, FDG-PET/CT scans showed good response to treatment with near complete resolution of FDG avidity. This rare case illustrates the rare pulmonary manifestation of extranodal lymphoma in a patient with Sjogren’s syndrome and emphasizes the value of FDG PET/CT in the initial staging and evaluation of response to treatment, which has not previously been published.

**Conflict of interest:**None declared.

## INTRODUCTION

Although extranodal non-Hodgkin’s lymphoma (NHL) represents up to one half of all cases of NHL, primary pulmonary lymphoma is only 3-4% of these cases, and less than 1% of all primary lung malignancies. In addition, Sjogren’s syndrome is a rare autoimmune disorder which affects only 3-4% of adults but these patients have a higher rate of NHL. There is no consensus on treatment or recommended follow-up, with current treatment options including surgery, chemotherapy and radiotherapy. This case illustrates the value of FDG-PET/CT in the detection and the follow-up of treatment of this rare disorder.

## CASE REPORT

**Figure 1:**
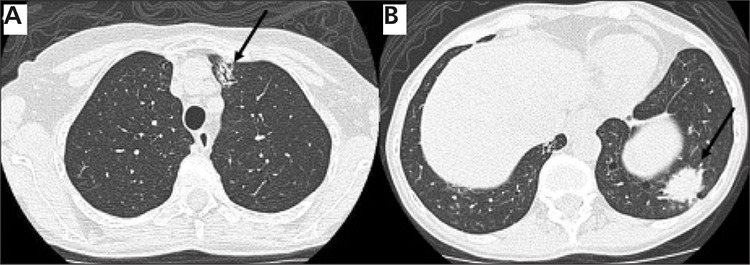
Axial images of a diagnostic CT scan on lung windowsettings demonstrate a spiculated 2x1.2 cm ill-defined opacity(arrow) in the anterior left upper lobe, and 2.6x2.2 cm opacity(arrow) in the posterior left lower lobe

**Figure 2:**
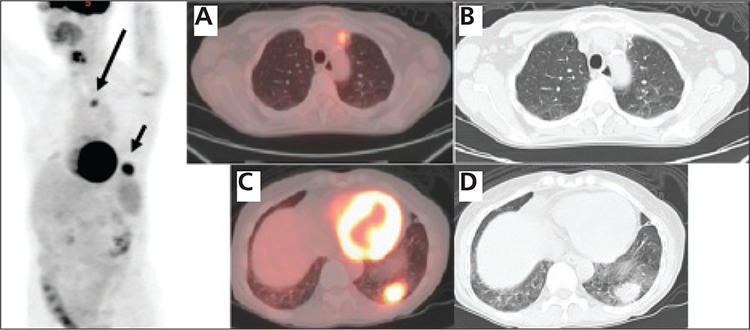
After biopsy of the left lower lobe lesionrevealed NHL, the initial staging PET/CT shows the increased FDGuptake associated with these pulmonary opacities in the left uppermedial chest (large arrow), and in the left lower lateral chest (smallarrow) on the 3D PET image. The fused axial image (A) and axial CTslice on lung window (B) demonstrate the increased FDG uptakeassociated with the opacity in the left upper lobe, with SUV max of5.2, which correlating the upper medial FDG uptake on 3D PETimage. The fused axial image (C) and axial CT slice (D) show the FDGavid parenchymal lesion in the left lower lobe measuring 2.9x2.2 cmwith SUV max of 10.2, correlating to the lower lateral FDG uptakeon 3D PET image. No other site of abnormal increased FDG uptakeis noted on the rest of the whole body PET images to suggest othersites of extranodal or nodal lymphomatous involvement

A 64- year old woman with chronic long standing history of Sjogren’s syndrome and chronic renal failure secondary to interstitial nephritis underwent evaluation for renal transplant surgery. Preoperative chest CT imaging revealed non-specific 2.6x2.2 cm opacity in the left lower lobe and a small opacity in the left upper lobe (Figure 1). The biopsy of the left lower lobe lesion revealed extranodal marginal B-cell non-Hodgkin’s lymphoma. A subsequent FDG-PET/CT scan showed intense FDG uptake in the left lower lobe opacity with SUV max of 10.2, as well as in the smaller ill-defined FDG-avid opacity in the left upper lobe with SUV max of 5.2 (Figure 2). The patient was treated with Rituximab for 12 weeks and was then placed on maintenance Rituximab. 

**Figure 3:**
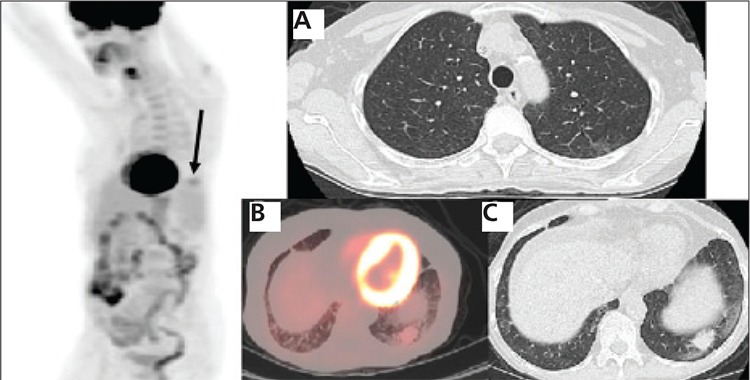
Two months after Rituxan therapy a restagingPET/CT shows a faint focus of abnormal increased FDG uptake inthe left lower lateral chest (arrow) and complete resolution of theFDG uptake in the upper medial chest on the 3D PET image. On theaxial CT image (A) the upper lobe lesion has completely resolved. Thefused axial image (B) and axial CT slice (C) show interval decrease inthe size and FDG uptake of the lower lobe lesion, to 2.4x1.7 cm withSUV max of 2.4, representing favorable response to treatment

**Figure 4:**
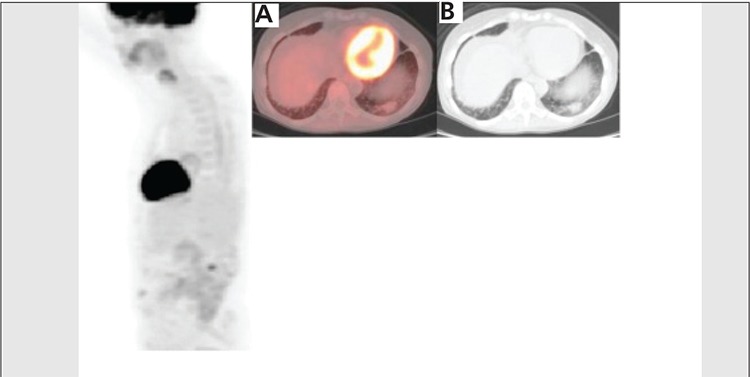
Six months after completion of therapy, 3DPET image shows interval near complete resolution of FDG uptake inthe left lower lateral chest. The fused axial image (A) and axial CTslice (B) demonstrate decrease in the size of left lower lobe lesionwhich has only subtle increased FDG uptake, the lesion at this timemeasured 2.4x1.4 cm with SUV max of 1.7, representing continuedfavorable response to treatment

A follow up FDG-PET/ CT two months after starting the initial Rituximab therapy showed interval slight decrease in the size and marked decrease in the metabolic activity of the left lower lobe opacity to SUV max of 2.4, and complete resolution of the left upper lobe opacity (Figure 3). Surveillance FDG-PET/CT 6 months after completion of initial Rituximab treatment showed continued decrease in the size and near complete interval resolution of metabolic activity of the left lower lobe pulmonary lesion, representing continued favorable response to treatment (Figure 4). 

## LITERATURE REVIEW AND DISCUSSION

Extranodal lymphoma refers to lymphomatous infiltration of anatomic sites other than the lymph nodes. Almost any organ can be affected by lymphoma, with the most common extranodal sites of involvement being the stomach, spleen, Waldeyer ring, central nervous system, lung, bone, and skin. The prevalence of extranodal involvement in non-Hodgkin lymphoma and Hodgkin disease has increased in the past decade. Imaging of tumor metabolism with FDG-PET has facilitated the identification of affected extranodal sites, even when CT has demonstrated no lesions. More recently, hybrid FDG-PET/CT has become the standard imaging modality for staging, follow-up, and treatment response assessment in patients with lymphoma and has proved superior to CT in these settings (1). 

Primary lymphoma of the lung is a rare presentation of extranodal NHL, representing only 0.5-1% of all primary pulmonary malignancies, less than 1% of all the cases of non-Hodgkin's lymphoma (NHL), and 3-4% of all the extranodal manifestations of NHL (2). Primary pulmonary lymphoma is diagnosed according to these 4 criteria: 1) Involvement of the lung, bronchus or both without evidence of mediastinal adenopathy or a mass, 2) No prior extrathoracic lymphoma diagnosis 3) No evidence of extrathoracic lymphoma or lymphatic leukemia at the time of primary lymphoma of the lung diagnosis 4) No extrathoracic disease for least three months after the initial diagnosis (3). This patient fulfilled all four criteria since the disease involved upper and lower lobes of the left lung, with no evidence of mediastinal adenopathy or mass, the patient had no prior history of extrathoracic lymphoma, and there was no evidence of extrathoracic lymphoma on the whole body PET imaging at the time of diagnosis or during the follow up.

Patients with Sjogren’s syndrome can develop opportunistic infections (4). Consideration in this setting should be made of the possibility of an isolated or superimposed opportunistic infection causing these lesions. Biopsy of the larger lesion was performed which revealed lymphoma only. Although PET/CT may be limited in the differential diagnosis of a coexisting opportunistic infection, the upper lobe lesion responded as did the lower lobe lesion to Rituximab treatment, indicating this finding was also most likely lymphoma. 

Sjogren’s syndrome is a systemic autoimmune disorder characterized by lymphocytic infiltrates in the exocrine organs which affects 3-4% of adults. The prevalence of any type of pulmonary involvement with Sjogren’s varies widely in the literature (5,6,7). The possible manifestations range from non-specific interstitial pneumonia, multiple lung cysts or bulla, to lymphoma (5). Since patients with Sjogren’s syndrome have a higher rate of non-Hodgkin lymphoma, detection and proper follow-up of treatment is important. About 5% will develop a lymphoid malignancy (as opposed to about 2% in the general population), most commonly salivary extranodal marginal zone B cell lymphoma and diffuse large B-cell lymphoma. Of those who develop primary pulmonary lymphoma, most are diagnosed with low-grade extra-nodal marginal zone B-cell lymphoma of the MALT type (8,9).

In the past two decades a marked increase has been reported in the incidence of extranodal lymphoma (10,11,12). Patients with primary extranodal disease without involvement of lymph node sites present at an earlier stage, whereas patients with NHL arising from lymph node sites tend to present with more advanced disease (13). Therefore, when extranodal lymphoma is diagnosed, it is important to determine whether the lesion is the primary site or not (14). Whole body FDG-PET/ CT is a promising diagnostic tool in the initial work up of primary extranodal disease. It could provide valuable information by excluding the presence of metabolically active nodal disease and confirm the diagnosis of primary extranodal lymphoma. 

There is no consensus on treatment, with current treatment options including surgery, chemotherapy and radiotherapy. FDG-PET/CT, is now widely utilized for response assessment of lymphoma after completion of therapy, for pre-treatment staging and increasingly for assessment of response during therapy (15,16,17). Particularly for response assessment at therapy conclusion, FDG-PET has been shown to be considerably more accurate than anatomical imaging because of its ability to distinguish between viable tumor and non viable post treatment findings. In this rare case of primary pulmonary lymphoma, PET/CT scan two months after the therapy showed resolution of upper lobe lesion and interval decrease in the size and FDG uptake of the lower lobe lesion. Surveillance PET/CT scan after 6 months of therapy showed near complete resolution of the FDG uptake in the left lower lobe lesion, confirming continued favorable treatment response.

FDG-PET/CT scan is becoming an indispensable tool in the initial staging, and evaluating response to treatment in patients with extranodal lymphoma and must be included in the clinical wok up of these patients. This rare case illustrates the usefulness of this modality in the initial detection and subsequent treatment of isolated pulmonary involvement with non Hodgkin’s` lymphoma in a patient with Sjogren’s syndrome.
